# A meta-analysis of ozone effect on tooth bleaching

**DOI:** 10.1038/s41598-021-92733-8

**Published:** 2021-06-23

**Authors:** Lia Dietrich, Marcelo Dias Moreira de Assis Costa, Cauane Blumenberg, Gustavo G. Nascimento, Luiz Renato Paranhos, Gisele Rodrigues da Silva

**Affiliations:** 1 School of Dentistry, Federal University of Uberlândia, Uberlândia, Brazil; 2 Federal University of Pelotas, Pelotas, RS Brazil; 3 Section of Periodontology, Department of Dentistry and Oral Health, Aarhus University, Aarhus, Denmark; 4 Department of Preventive and Social Dentistry, School of Dentistry, Federal University of Uberlândia, Uberlândia, Brazil; 5 Department of Operative Dentistry and Dental Materials, Dental School, Federal University of Uberlândia, Uberlândia, MG Brazil

**Keywords:** Health care, Medical research

## Abstract

This systematic review assessed the effectiveness of ozone (O_3_) in the color change of in-office tooth bleaching in vital teeth (TB) and the sensitivity control. Only randomized controlled clinical trials were included. Seven databases were used as primary search sources, and three additional sources were searched to capture the "grey literature" partially. The JBI tool was used to assess the risk of bias. TB was assessed using the ΔELab color change metric comparing tooth color pre- and post-bleaching. We meta-analyzed the ΔELab estimates per method and calculated the absolute standardized mean difference using random-effect models. The GRADE approach assessed the certainty of the evidence. The ΔELab estimates ranged from 1.28 when the O_3_ was used alone to 6.93 when combined with hydrogen peroxide (HP). Two studies compared O_3_ and HP alone, but their TB was similar (SMD = − 0.02; 95%CI: − 0.54; 0.49). The bleaching effectiveness for the combination of O_3_ + HP compared to HP was similar (SMD = 0.38; 95%CI: − 0.04; 0.81). Thus, based on the available literature, our findings suggest that O_3_ is not superior to the conventional technique using HP on the change of tooth color. The O_3_ did not present sensitivity when used alone. When O_3_ was used in combination with HP, patients reported hypersensitivity only when O_3_ was applied before HP, i.e., no sensitivity was perceived when O_3_ was applied after HP.

## Introduction

Tooth bleaching of vital teeth has become popular over the last decades despite the adverse effects associated with the procedure, such as tooth sensitivity^1–8^, gingival irritation^2,6,8^ morphology changes on the enamel surface^9^, the inflammatory response of the pulp tissue^2,10–13^, reduction of the metabolism and cell viability^14^, changes in vascular permeability^15^, increased marginal micro infiltration in the tooth/restoration interface^2^, and microhardness reduction of restorative materials^16^. Besides these adverse effects, studies have shown that the chemical components of bleaching gels may have cytotoxic and carcinogenic effects^2,8,17^.

The most common adverse effect after tooth bleaching therapy is tooth sensitivity, with a mean prevalence of 70% in patients during and after the procedure^5^. Such sensitivity may be related to the use of bleaching gels, which are made of hydrogen peroxide (HP)^1,5,6,12^. This material has low molecular weight and can spread through enamel and dentin, promoting tooth bleaching but potentially damaging pulp cells^11^. The free radicals formed by the dissociation of HP are mainly responsible for the toxicity of this compound because its oxidative reactions may cause damage to odontoblasts and decrease their metabolic activity^2,3,5^.

Ozone (O_3_) is a natural gas formed by three oxygen atoms, and it has been used for medical therapies since World War I^18,19^. Currently, health professionals use ozone therapy^20^ for the treatment of several pathologies due to its high oxidation power, immune response, circulatory stimulation, analgesic and anti-inflammatory properties, and parasitological effect^21–23^. In dentistry, O_3_ effectively controls infections caused by viruses, protozoa, fungi, and bacteria^18,21^. Moreover, it seems to promote tissue repair and healing processes^24^, prevention of dental caries^22,23,25^, remineralization of the tooth surface^22,25^, treatment of oral ulcers^22^, treatment of gingivitis and periodontitis^24^, pain control^22,25,26^, endodontic treatment^27^, halitosis^19,21^, temporomandibular disorders^19,21^, complementary treatment of non-carious cervical lesions and tooth sensitivity^28–30^, and tooth bleaching^28–32^.

Using O_3_ for tooth bleaching is safe in conditions in which diffusion is an important factor, such as in hard dental tissues, as it works on their organic substances and can be used, for instance, to reduce tetracycline staining^28^. However, the effectiveness of ozone therapy in tooth bleaching may depend on the application time, bleaching gel concentration, and gas flow rate^33^. There is still no consensus in the literature on the best usage protocol for O_3_ and HP for tooth bleaching. Thus, this systematic review aims to evaluate whether O_3_ can improve the clinical performance of tooth bleaching in vital teeth. The authors worked with the following hypotheses: (1) O_3_ can promote color change in tooth bleaching better than HP, (2) O_3_ associated with HP accelerates the effect of color change in tooth bleaching, and (3) O_3_ reduces tooth sensitivity caused by tooth bleaching.

## Methods

### Protocol and registration

This systematic review followed the recommendations listed in the Preferred Reporting Items for Systematic Reviews and Meta-Analyses Protocols (PRISMA)^34^ and the Cochrane guidelines^35^. The protocol of this systematic review was registered in the International Prospective Register of Systematic Reviews (PROSPERO), under number CRD42018099190 (https://www.crd.york.ac.uk/prospero/).

### Study design and eligibility criteria

The systematic review aimed to answer the following guiding question, based on the PICO strategy: Do patients treated with tooth bleaching in vital teeth (patients) with ozone therapy (intervention) have improved clinical results of color change and tooth sensitivity (outcome) when compared to the conventional treatment with HP (control)?

Only randomized clinical trials (RCTs) reporting the use of O_3_ alone or combined with HP gel as one of their study groups for tooth bleaching were included. There was no restriction of year, language, and publication status.

The exclusion criteria were: 1) studies not related to the topic; 2) reviews, observational studies, letters to the editor/editorials, personal opinions, books/book chapters, reports, conference abstracts, and theses; 3) laboratory studies; and 4) case reports and case series.

### Sources of information, search and study selection

Cochrane, Embase, LILACS, PubMed, SciELO, Scopus, and Web of Science were the primary databases used for searching the studies. The OATD, OpenThesis, and OpenGrey databases were used to partially capture the "grey literature". The Medical Subject Headings (MeSH), Health Sciences Descriptors (DeCS), and Embase Subject Headings (Emtree) resources were used for selecting the keywords. The Boolean operators "AND" and "OR" were used to enhance the research strategy through several combinations (Table 1). A manual search was also performed through a systematized analysis of the references of the studies that had previously achieved the eligibility step. The search was performed in May 2020. The results obtained from the primary databases were initially exported to EndNote Web (Clarivate Analytics, Philadelphia, USA), excluding the duplicates. Then, they were exported to Microsoft Word (Microsoft Ltd, Washington, USA) as well as the results obtained in the grey literature, in which the remaining duplicates were removed manually.

**Table 1:** Strategies for database search.

Database	Search strategy (May, 2020)
PubMed http://www.ncbi.nlm.nih.gov/pubmed	(("Bleaching, Tooth" OR "Teeth Whitening" OR “Hypersensitivity” OR "Whitening, Teeth" OR “Dentin Sensitivity” OR "Tooth Whitening" OR "Whitening, Tooth" OR "Teeth Bleaching" OR "Bleaching, Teeth" OR “Agents, Tooth Bleaching" OR “Colour Change” OR "Bleaching Agents, Tooth" OR "Teeth Whitening Agents" OR “Agents, Teeth Whitening” OR “Whitening Agents, Teeth” OR “Tooth Whitening Agents” OR “Agents, Tooth Whitening” OR “Whitening Agents, Tooth” OR “Teeth Bleaching Agents” OR “Agents, Teeth Bleaching” OR “Bleaching Agents, Teeth” OR “Agents, Bleaching” OR “Whitening Agents” OR “Agents, Whitening” OR “Sensitivity”) AND (“Ozone” OR “Ozonotherapy” OR “Ozone Therapy” OR “O_3_”))
Scopus http://www.scopus.com	( TITLE-ABS-KEY ( ( "Tooth Whitening" OR "Sensitivity" OR "Teeth Whitening Agents" OR "Tooth Bleaching" OR "Hypersensitivity" OR "Tooth Whitening Agents" OR "Color Change" OR "Dentin Sensitivity" OR "Bleaching Agents" ) ) OR TITLE-ABS-KEY ( ( "Bleaching Agents, Tooth" OR "Color Change" OR "Tooth Whitening" OR "Sensitivity" OR "Hypersensitivity" OR "Tooth Whitening Agents" OR "Tooth Bleaching" ) ) AND TITLE-ABS-KEY ( ( "Ozone" OR "Ozonotherapy" OR "Ozone Therapy" OR "O3" ) ) )
LILACS http://lilacs.bvsalud.org/	tw:("Bleaching, Tooth" OR "Teeth Whitening") AND (“Ozone” OR “Ozonotherapy”) AND (db:("LILACS"))
	tw:("Whitening, Teeth" OR "Tooth Whitening") AND (“Ozone” OR “Ozonotherapy”) AND (db:("LILACS"))
	tw:("Whitening, Tooth" OR "Teeth Bleaching") AND (“Ozone Therapy” OR “O3”) AND (db:("LILACS"))
	tw:("Bleaching, Teeth" OR "Agents, Tooth Bleaching”) AND (“Ozone Therapy” OR “O3″) AND (db:("LILACS"))
	tw:("Bleaching Agents, Tooth" OR "Teeth Whitening Agents") AND (“Ozone” OR “Ozonotherapy”) AND (db:("LILACS"))
	tw:(“Agents, Teeth Whitening” OR “Whitening Agents, Teeth”) AND (“Ozone” OR “Ozonotherapy”) AND (db:("LILACS"))
	tw:(“Tooth Whitening Agents” OR “Agents, Tooth Whitening”) AND (“Ozone” OR “Ozonotherapy”) AND (db:("LILACS"))
	tw:(“Tooth Whitening Agents” OR “Hypersensitivity”) AND (“Ozone” OR “Ozonotherapy”) AND (db:("LILACS"))
	tw:("Bleaching, Tooth" OR "Color Change") AND (“Ozone” OR “Ozonotherapy”) AND (db:("LILACS"))
	tw:("Sensitivity" OR "Teeth Bleaching") AND (“Ozone Therapy” OR “O3”) AND (db:("LILACS"))
	tw:("Whitening, Tooth" OR "Dentin Sensitivity") AND (“Ozone Therapy” OR “O3”) AND (db:("LILACS"))
	tw:(“Bleaching Agents, Teeth” OR “Agents, Bleaching”) AND (“Ozone Therapy” OR “O3”) AND (db:("LILACS"))
	tw:(“Whitening Agents” OR “Agents, Whitening”) AND (“Ozone” OR “Ozone Therapy”) AND (db:("LILACS"))
SciELO http://www.scielo.org/	Bleaching, Tooth AND Ozone
	Whitening Agents AND Ozone
	Agents, Whitening AND Ozone
	Bleaching Agents, Teeth AND Ozone
	Agents, Tooth Whitening AND Ozone
	Sensitivity AND Ozone
	Color Change AND Ozone
	Hypersensitivity AND Ozone
	Dentin Sensitivity AND Ozone
	Tooth Whitening Agents AND Ozone
	Bleaching Agents, Tooth AND Ozone
	Teeth Whitening Agents AND Ozone
	Bleaching Teeth AND Ozone
	Teeth Bleaching AND Ozone
	Teeth Whitening AND Ozone Therapy
	Whitening Teeth AND Ozone therapy
	Whitening Tooth AND Ozone
	Blanqueamiento de dientes AND Ozono *[Spain]*
	Blanqueadores AND Ozono *[Spain]*
	Blanqueadores dentales AND Ozono *[Spain]*
	Blanqueo de Diente AND Ozono *[Spain]*
	Blanqueo de Dientes AND Ozono *[Spain]*
	Sensibilidad AND Ozono *[Spain]*
	Hipersensibilidad AND Ozono *[Spain]*
	Sensibilidad a la Dentina AND Ozono *[Spain]*
	Agentes Blanqueadores Dentales AND Ozono *[Spain]*
Embase http://www.embase.com	('bleaching, tooth' OR 'teeth whitening' OR 'hypersensitivity' OR 'whitening, teeth' OR 'dentin sensitivity' OR 'tooth whitening' OR 'whitening, tooth' OR 'teeth bleaching' OR 'bleaching, teeth' OR 'agents, tooth bleaching' OR 'color change' OR 'bleaching agents, tooth' OR 'teeth whitening agents' OR 'agents, teeth whitening' OR 'whitening agents, teeth' OR 'tooth whitening agents' OR 'agents, tooth whitening' OR 'whitening agents, tooth' OR 'teeth bleaching agents' OR 'agents, teeth bleaching' OR 'bleaching agents, teeth' OR 'agents, bleaching' OR 'whitening agents' OR 'agents, whitening' OR 'sensitivity') AND ('ozone' OR 'ozonotherapy' OR 'ozone therapy' OR 'o3')
Web Of Science http://apps.webofknowledge.com/	(("Bleaching, Tooth" OR "Teeth Whitening" OR “Hypersensitivity” OR "Whitening, Teeth" OR “Dentin Sensitivity” OR "Tooth Whitening" OR "Whitening, Tooth" OR "Teeth Bleaching" OR "Bleaching, Teeth" OR “Agents, Tooth Bleaching" OR “Color Change” OR "Bleaching Agents, Tooth" OR "Teeth Whitening Agents" OR “Agents, Teeth Whitening” OR “Whitening Agents, Teeth” OR “Tooth Whitening Agents” OR “Agents, Tooth Whitening” OR “Whitening Agents, Tooth” OR “Teeth Bleaching Agents” OR “Agents, Teeth Bleaching” OR “Bleaching Agents, Teeth” OR “Agents, Bleaching” OR “Whitening Agents” OR “Agents, Whitening” OR “Sensitivity”) AND (“Ozone” OR “Ozonotherapy” OR “Ozone Therapy” OR “O_3_”))
Cochrane https://www.cochranelibrary.com/search	("Bleaching, Tooth" OR "Teeth Whitening" OR “Hypersensitivity” OR "Whitening, Teeth" OR “Dentin Sensitivity” OR "Tooth Whitening" OR "Whitening, Tooth" OR "Teeth Bleaching" OR "Bleaching, Teeth" OR “Agents, Tooth Bleaching" OR “Color Change” OR "Bleaching Agents, Tooth" OR "Teeth Whitening Agents" OR “Agents, Teeth Whitening” OR “Whitening Agents, Teeth” OR “Tooth Whitening Agents” OR “Agents, Tooth Whitening” OR “Whitening Agents, Tooth” OR “Teeth Bleaching Agents” OR “Agents, Teeth Bleaching” OR “Bleaching Agents, Teeth” OR “Agents, Bleaching” OR “Whitening Agents” OR “Agents, Whitening” OR “Sensitivity”) AND (“Ozone” OR “Ozonotherapy” OR “Ozone Therapy” OR “O3”)
OpenGrey http://www.opengrey.eu/	(("Bleaching, Tooth" OR "Teeth Whitening" OR “Hypersensitivity” OR "Whitening, Teeth" OR “Dentin Sensitivity” OR "Tooth Whitening" OR "Whitening, Tooth" OR "Teeth Bleaching" OR "Bleaching, Teeth" OR “Agents, Tooth Bleaching" OR “Colour Change” OR "Bleaching Agents, Tooth" OR "Teeth Whitening Agents" OR “Agents, Teeth Whitening” OR “Whitening Agents, Teeth” OR “Tooth Whitening Agents” OR “Agents, Tooth Whitening” OR “Whitening Agents, Tooth” OR “Teeth Bleaching Agents” OR “Agents, Teeth Bleaching” OR “Bleaching Agents, Teeth” OR “Agents, Bleaching” OR “Whitening Agents” OR “Agents, Whitening” OR “Sensitivity”) AND (“Ozone” OR “Ozonotherapy” OR “Ozone Therapy” OR “O_3_”))
OpenThesis http://www.openthesis.org/	("Bleaching Tooth" OR "Dentin Sensitivity" OR "Teeth Whitening" OR "Whitening Teeth" OR "Hypersensitivity" OR "Tooth Whitening" OR "Whitening Tooth" OR "Color Change" OR "Teeth Bleaching" OR "Sensitivity" OR "Bleaching Teeth" OR "Tooth Bleaching Agent") AND ("Ozone" OR "Ozonotherapy" OR "Ozone Therapy" OR "O3") AND ("Clinical Trials" OR "Clinical Studies" OR "Clinical Investigation" OR "Clinical Research" OR "Clinical Evidence")
Open Access Theses and Dissertations (OATD) https://oatd.org/	(("Bleaching, Tooth" OR "Teeth Whitening" OR "Whitening, Teeth" OR "Tooth Whitening" OR "Whitening, Tooth" OR "Teeth Bleaching" OR "Bleaching, Teeth" OR "Agents, Tooth Bleaching") AND (“Ozone” OR “Ozonotherapy” OR “Ozone Therapy” OR “O3”))

Before selecting the studies, a calibration exercise was performed among the reviewers. Subsequently, exclusion by titles (first phase), by abstracts (second phase), and by reading the full articles (third phase) was performed. All phases were independently evaluated by two evaluators (LD and MDMAC), and, in case of doubt or disagreement, a third evaluator (LRP) was always consulted to make a final decision.

### Data collection

Prior to data extraction, both reviewers (LD and MDMAC) were calibrated by extracting the data from one article and comparing it with the third reviewer, with expertise in dental bleaching and systematic reviews. The reviewers extracted the following information: identification of the study (author, year, location), sample characteristics (number of patients, distribution by sex, and average age), characteristics of sample collection and processing (groups, materials used, application time and follow-up, teeth assessed), specific results: quantification of 1) color change using ΔE_Lab_, CIELab (a, b, and L) and 2) dentin sensitivity using the Visual Analogue Scale (VAS). We evaluated whether the studies respected the ethical criteria for the research development according to the current law in the countries of origin, whether the previous signature of the consent form was collected, whether the CONSORT was used as a guideline, and whether the studies were registered in databases of clinical trials. Lastly, the analysis and the results (bleaching effectiveness, O_3_ effectiveness in bleaching, O_3_ influence on sensitivity) were analyzed. In case of doubt regarding the data presented in the results of the studies, the authors were contacted.

### Risk of individual bias of the studies

The JBI Manual for Evidence Synthesis^36^ (LD and MDMAC) assessed each domain independently regarding the potential risk of bias, as recommended by the PRISMA statement^34^.

Each study was categorized according to the percentage of positive answers to the questions. The risk of bias was considered “High” when the study obtained 49% or less “yes” answers, “Moderate” when the study obtained 50% to 69% of “yes” answers, and “Low” when the study reached more than 70% of “yes” score.

### Summary measures and meta-analysis

In order to assess bleaching effectiveness, the CIE_Lab_ (L, a, b) system for measuring color difference was explored. From these data, the delta E (ΔE_Lab_), which measures the color change between the pre- and post-bleaching periods for all bleaching methods, was calculated. As some studies did not provide the ΔE_Lab_, calculation, the estimate was calculated by the CIE76 formula: ${\Delta E}_{Lab}=\sqrt{\Delta {L}^{2}+\Delta {a}^{2}+\Delta {b}^{2}}$^37^.

A meta-analysis with a random-effects model was performed using the Stata 16.0 software (StataCorp., College Station, TX, USA). The ΔE_Lab_ estimates from the different methods were compared by absolute standardized mean differences (SMD) to compare the bleaching effectiveness. We did not meta-analyzed the VAS measures since only one study^28^ would be eligible, since the remaining studies^29,30^ did not show any variability on the VAS scale comparing comparing the pre- and post-bleaching periods.

### Certainty of evidence collection

The certainty of evidence and strength of recommendation were assessed with the Grading of Recommendation, Assessment, Development, and Evaluation (GRADE) tool^38^. The GRADE pro-GDT software (http://gdt.guidelinedevelopment.org) was used for summarizing the results. This assessment was based on study design, methodological limitations, inconsistency, indirect evidence, imprecision, and other considerations. The quality of evidence was characterized as high, moderate, low, or very low^38^.

## Results

### Study selection

**Figure 1:**
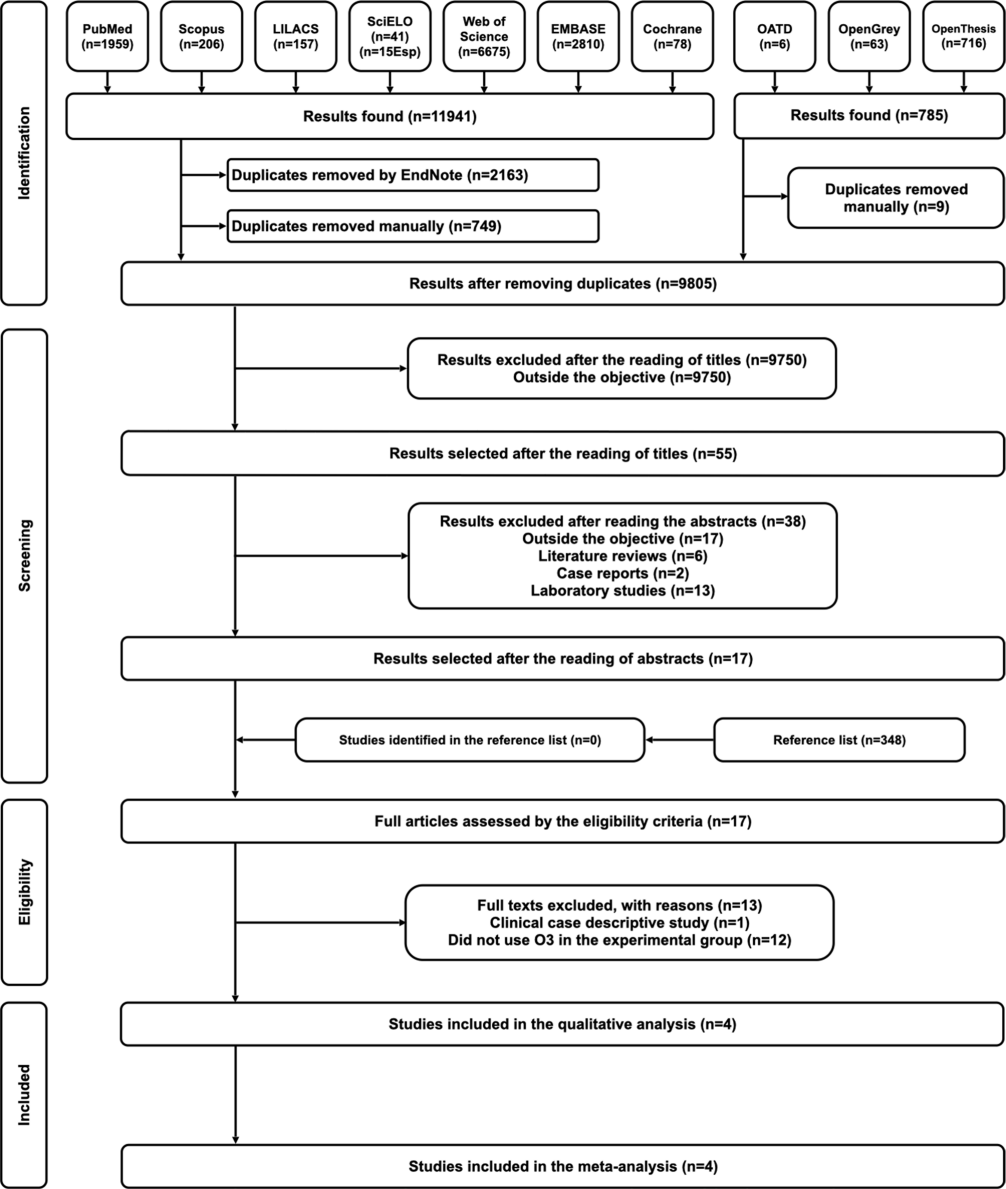
Flowchart of the process of literature search and selection, adapted from the PRISMA statement.

A total of 12,703 results were found in ten electronic databases, including “gray literature”, in the first phase of the study selection. After analysis, only 17 studies were eligible for full-text analysis. The references of the 17 potentially eligible studies were evaluated, and no additional articles were selected. After reading the entire text, 13 studies did not meet the inclusion criteria and were eliminated: twelve were literary reviews, and one was a congress summary. Thus, four studies were included in this review (Fig. 1).

### Characteristics of eligible studies

The studies were published between 2016 and 2018 and were performed in Jordan^28–30^ and Turkey^32^. All studies^28–30,32^ respected the ethical criteria for research development recommended in each country of origin, applying a consent form for all volunteers participating in the study. Only one study^30^ mentioned using the CONSORT as a guideline, and none of the studies clarified whether they were registered in clinical trial databases.

The total sample included 129 patients treated with tooth bleaching, with 57 in the control group treated with 38% HP and 72 treated with bleaching with O_3_. From the latter, 29 were treated with O_3_ alone, while 43 were treated with O_3_ associated with HP. From all patients, 77 were women, and 52 were men. The age of the patients in each study ranged between 24 and 50 years^28^, 20 to 35 years^32^, 20 to 35 years^30^, and 19 and 33 years^29^.

All studies used methods of color analysis, as follows: Spectrophotometer^32^, Colorimeter Konica^28–30^, and Vita Classical^28–30^. The color assessment was registered only in the maxillary dental arch at the following times: initial (before bleaching started)^28–30,32^, after bleaching (24 h)^28–30^, and immediately after bleaching and 48 h later^32^. Table 2 shows detailed characteristics of the eligible studies.

**Table 2:** Summary of the main characteristics of the eligible studies.

Authors (Year)	Location	Method of color analysis	Average age of the individual and standard deviation (years)	Number of individuals eligible for analyses (% male)	Groups/materials	Initial color/tooth assessed	Result assessed		Follow-up	Effect of ozone on bleaching	Effect of ozone on sensitivity
							Color change	Tooth sensitivity			
Al-Omiri et al. (2016)^28^	Amman, Jordan	Colorimeter Konica-Minolta CR-400 (Minolta Inc, Osaka, Japan)	27 ± 5	26 (50)	I—H_2_O_2_ 38%^a^ (20 min) + O_3_^b^ (60 s) II—H_2_O_2_ 38%^a^ (20 min)	A3 or darker/anterior teeth	Vita Classical ∆L, ∆a, ∆b	VAS	24 h after bleaching	positive	positive
Aykut-Yetkiner et al. (2017)^32^	Izmir, Turkey	Spectrophotometer (Vita Easyshade, Vident, Brea, CA, USA)	36.2 ± 8.7	26 (7.7)	I—H_2_O_2_ 40%^c^ (40 min) II—O_3_^d^ (40 min)	n.r./upper incisors	∆E, ∆L, ∆a, ∆b	Not applicable	Immediately and 48 h after bleaching	positive	Not applicable
Al-Omiri et al. (2018)^30^	Amman, Jordan	Colorimeter Konica-Minolta CR-400 (Minolta Inc, Osaka, Japan)	25 ± 4	45 (46.7)	I—O_3_^e^ (60 s) + H_2_O_2_ 38%^a^ (20 min) II—H_2_O_2_ 38%^a^ (20 min) + O_3_^e^ (60 s) III—H_2_O_2_ 38%^a^ (20 min)	A3 or darker/anterior teeth	Vita Classical L, ∆a, ∆b	VAS	24 h after bleaching	positive	Group I – no Group II—positive
Al-Omiri and others (2018)^29^	Amman, Jordan	Colorimeter Konica-Minolta CR-400 (Minolta Inc, Osaka, Japan)	23 ± 5	32 (50)	I—O_3_^e^ (60 s) II—H_2_O_2_ 38%^a^ (20 min)	n.r./anterior teeth	Vita Classical L, ∆a, ∆b	VAS	24 h after bleaching	positive	positive

*n.r.* not reported or not applicable; *∆E* color change variation according to the CIELAB system; *∆L* variation of the black/white matrix axis in the CIELAB system; *∆a* variation of the red/green matrix axis in the CIELAB system; *∆b* variation of the yellow/blue matrix axis in the CIELAB system; *VAS* Visual Analogue Scale designed as a 10-cm horizontal line with the words "no pain" in one end and "worst pain" in the opposite end; O_3_: ozone; H_2_O_2_: hydrogen peroxide.

^a^White 38% BMS, Dental BMS.

^b^HealOzone X4, KaVo Dental, Biberach.

^c^Opalescence PF, Ultradent products.

^d^Oxonytron OZ, Mio International.

^e^The HealOzone X4 device, Curozone.

### Risk of individual bias of the studies

Two eligible studies^28,32^ had a “moderate” risk of bias or methodological quality while two studies^28,30^ “low” risk of bias. Table 3 shows detailed information on the risk of bias of the studies included. Item 1 was marked as “Unclear” in two studies because the randomization method was not explicit^28,32^. Item 2 was marked as “Unclear” in one study because it did not describe the steps followed for hiding the sequence until attributing the interventions^30^, and marked as “No” in three studies^28,29,32^ because randomization was not explained. As for item 3, two studies were marked as “No” because they did not describe the baseline^29,32^. In item 4, two studies did not inform about participants blinding^29,32^. All four studies were marked as “No” in item 5 because they did not blind the operators^28–30,32^. In item 6, only one study was marked as “No” because it did not blind the evaluator from the result^32^. All studies were marked as “Not applicable” in item 9 because there was no participant dropout and the follow-up time was rather short^28–30,32^.

**Table 3:** Risk of bias assessed by the JBI Manual for Evidence Synthesis. The risk of bias was classified as high when the study reached up to 49% of "yes" score, moderate when the study reached from 50 to 69% of "yes" score, and low when the study reached more than 70% of "yes" score.

Authors	Q.1	Q.2	Q.3	Q.4	Q.5	Q.6	Q.7	Q.8	Q.9	Q.10	Q.11	Q.12	Q.13	% yes/risk
Al-Omiri et al. (2016)^28^	U	–	√	√	–	√	√	√	N/A	√	√	√	√	75%/low
Aykut-Yetkiner et al. (2017)^32^	U	–	–	–	–	–	√	√	N/A	√	√	√	√	50%/ moderate
Al-Omiri et al. (2018)^30^	√	U	√	√	–	√	√	√	N/A	√	√	√	√	83%/low
Al-Omiri and others (2018)^29^	√	–	–	–	–	√	√	√	N/A	√	√	√	√	66%/ moderate

Q.1—Was true randomization used for assigning the participants to treatment groups? Q.2—Was allocation to groups concealed? Q.3—Were treatment groups similar at the baseline? Q.4—Were participants blind to treatment assignment? Q.5—Were those delivering treatment blind to treatment assignment? Q.6—Were outcome evaluators blind to treatment assignment? Q.7—Were treatment groups treated identically other than the intervention of interest? Q.8—Was follow-up complete and, if not, were differences between groups in terms of their follow-up adequately described and analyzed? Q.9—Were participants analyzed in the groups to which they were randomized? Q.10—Were outcomes measured equally for treatment groups? Q.11—Were outcomes measured in a reliable way? Q.12 -Was appropriate statistical analysis used? Q.13—Was the trial design appropriate for the topic and were any deviations from the standard RCT design considered in the conduct and analysis? / √ Yes; – No; *U* Unclear; *N/A* not applicable.

### Specific results of the eligible studies

One of the studies assessed the result of color change in tooth bleaching immediately after applying the products and 48 h later^32^, while the remaining studies performed this assessment 24 h after the procedure^28–30^. These three studies also measured tooth sensitivity after bleaching^28–30^.

In all studies and all experimental groups, the results of color change in tooth bleaching were positive for whitening the teeth, changing the initial color. Bleaching with O_3_ presented statistically similar results to the groups using HP in the studies^28–30^.

Bleaching with HP (control group) induced tooth sensitivity in all studies analyzed, and ozone therapy applied alone or after the use of HP was able to eliminate the painful symptomatology and reduce the time of gel application without changing bleaching effectiveness. The ΔE_Lab_ was pre-informed in only one study^32^ and calculated for the others using the CIE76 formula, as mentioned by Gaurav^37^.

### Synthesis of results and meta-analysis

Table 4 shows the results of color change and tooth sensitivity for each study. Although all groups achieved positive ΔE_Lab_ estimates, indicating effective bleaching, there was high variability between study results. The ΔE_Lab_ estimates ranged from 1.28 when the ozone therapy was used alone to 6.93 when combined with HP.

**Table 4:** Color difference results of the eligible studies and dentin sensitivity.

Author (year)	Groups	N	∆L	∆a	∆b	∆E	∆VAS
Al-Omiri et al. (2016)^28^	H_2_O_2_ 38%^a^ (20 min) + O_3_^b^ (60 s)	13	4.70 (1.76)	− 1.50 (0.83)	− 4.86 (1.63)	6.93 (5.97)	0.00 (0.00)
	H_2_O_2_ 38%^a^ (20 min)	13	1.78 (2.27)	− 0.73 (0.98)	− 2.81 (2.28)	3.41 (8.05)	1.72 (0.50)
Aykut-Yetkiner et al. (2017)^32^	H_2_O_2_ 40%^c^ (40 min)	13	0.82 (1.72)	0.22 (0.38)	1.43 (1.50)	1.66 (5.40)	n.r
	O3^d^ (40 min)	13	0.57 (1.92)	0.24 (0.71)	1.12 (2.85)	1.28 (8.42)	n.r
Al-Omiri et al. (2018)^30^	O_3_^e^ (60 s) + H_2_O_2_ 38%^a^ (20 min)	15	3.42 (1.82)	− 0.31 (0.82)	− 4.73 (1.56)	5.85 (5.96)	3.20 (0.57)
	H_2_O_2_ 38%^a^ (20 min) + O_3_^e^ (60 s)	15	3.08 (2.15)	− 0.65 (1.02)	4.27 (2.18)	5.30 (7.71)	0.00 (0.00)
	H_2_O_2_ 38%^a^ (20 min)	15	1.45 (2.09)	− 0.54 (0.96)	− 2.66 (2.43)	3.08 (8.01)	1.60 (0.46)
Al-Omiri and others (2018)^29^	O_3_^e^ (60 s)	16	1.38 (1.87)	− 0.55 (0.85)	− 2.82 (1.57)	3.19 (6.09)	0.00 (0.00)
	H_2_O_2_ 38%^a^ (20 min)	16	1.62 (2.00)	− 0.61 (0.92)	− 2.63 (2.34)	3.15 (7.68)	1.31 (0.40)

*n.r*. not reported; *∆E* color change variation according to the CIELAB system; *∆L* variation of the black/white matrix axis in the CIELAB system; *∆a* variation of the red/green matrix axis in the CIELAB system; *∆b* variation the of yellow/blue matrix axis in the CIELAB system; *VAS* Visual Analogue Scale designed as a 10-cm horizontal line with the words “no pain” in one end and “worst pain” in the opposite end; *O*_*3*_ ozone; *H*_*2*_*O*_*2*_ Hydrogen peroxide.

**Figure 2:**
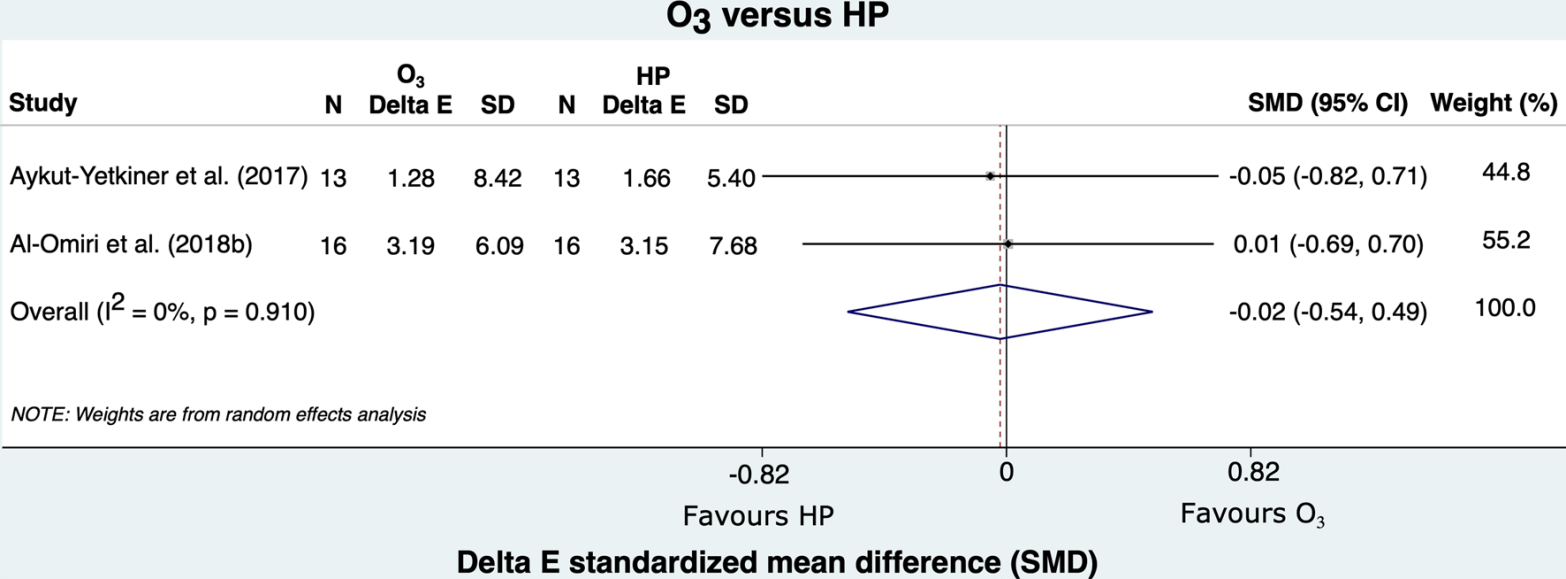
Forest plot of color change comparing group O_3_ to group HP.

**Figure 3:**
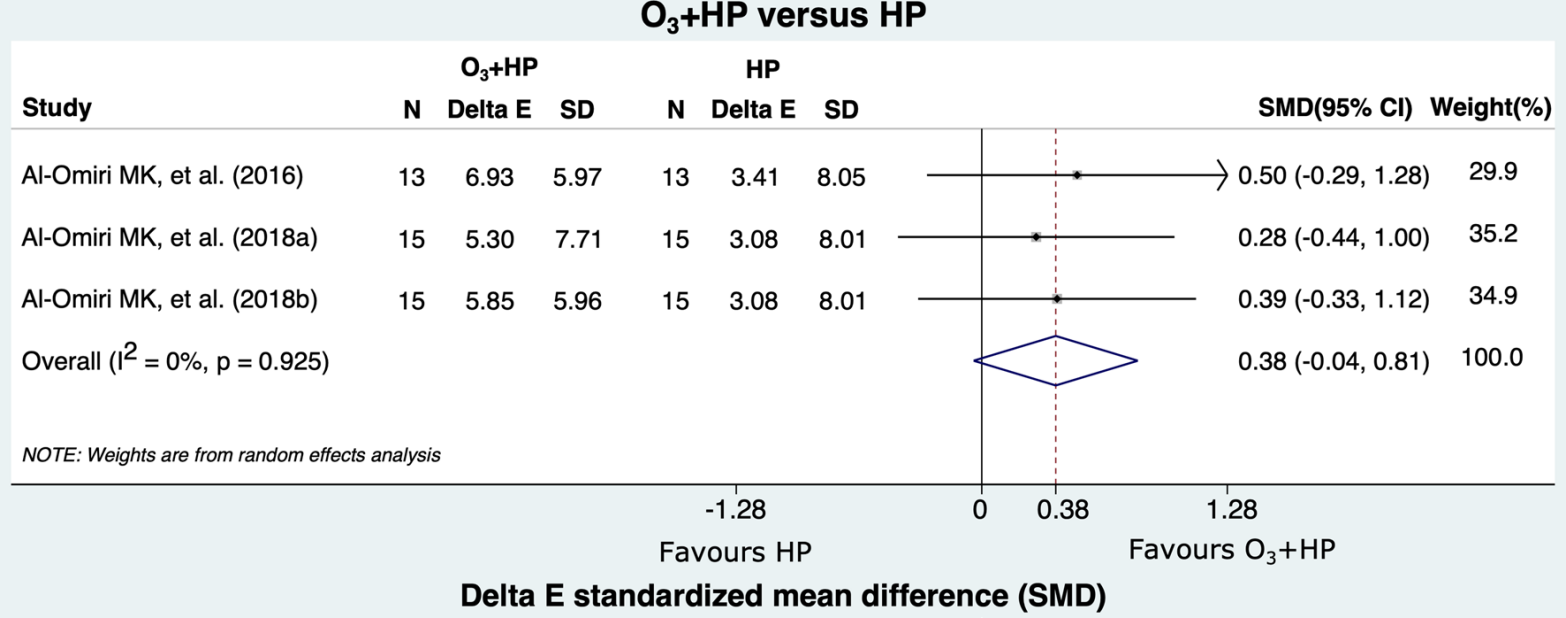
Forest plot of color change comparing group HP + O_3_ to group HP.

Figure 2 shows the comparison between the bleaching effectiveness of ozone therapy and HP alone. Only two studies compared these agents, which achieved a similar bleaching effectiveness (SMD = − 0.02; 95%CI: − 0.54; 0.49). On a similar note, comparing the effectiveness of O_3_ and HP combined to HP alone showed that bleaching effectiveness was also similar between the techniques (SMD = 0.38; 95%CI: − 0.04; 0.81) (Fig. 3).

Regarding tooth sensitivity, ΔVAS scores comparing pre- and post-bleaching periods ranged from 0.0 to 3.2. The highest sensitivity score among all studies (ΔVAS = 3.20) was reported in the group treated with O_3_ followed by HP. Two other studies using a similar combination but applying HP before the ozone reported no tooth sensitivity (Table 4).

### Certainty of evidence

The GRADE tool assessed two outcomes (Bleaching effectiveness—O_3_ vs. H_2_O_2_ and Bleaching effectiveness O_3_ + H_2_O_2_ vs. H_2_O_2_). All outcomes were categorized as a very low level of certainty, which means the true effect is likely to be substantially different from the estimated effect. The two outcomes were downgraded in two levels due to risk of bias (limitations in randomization and blindness), imprecision (wide credible intervals and a low number of participants), and publication bias (three out of four articles were performed by the same research group). Table 5 shows more details for each outcome.

**Table 5:** Summary of findings by the Grading of Recommendations Assessment, Development, and Evaluation (GRADE) for the outcomes of the systematic review and meta-analysis.

Quality assessment							Summary of results		
Number of studies	Study design	Risk of bias	Inconsistency	Indirectness	Imprecision	Others considerations	Number of participants	Effect	General quality
								SMD (95%CI)	
**Outcome 1: Bleaching effectiveness—O**_**3**_** vs H**_**2**_**O**_**2**_									
2	RCT	Serious^1^	Not serious	Not serious	Serious^2^	Publication bias strongly suspected^3^	58	− 0.02 (− 0.54, 0.49)	⨁ VERY LOW
**Outcome 2: Bleaching effectiveness—O**_**3**_** + H**_**2**_**O**_**2**_** vs H**_**2**_**O**_**2**_									
3	RCT	Serious^1^	Not serious	Not serious	Serious^2^	Publication bias strongly suspected^3^	103	0.38 (− 0.04, 0.81)	⨁ VERY LOW

GRADE Working Group grades of evidence.

High certainty: We are very confident that the true effect lies close to that of the estimate of the effect.

Moderate certainty: We are moderately confident in the effect estimate: The true effect is likely to be close to the estimate of the effect, but there is a possibility that it is substantially different.

Low certainty: Our confidence in the effect estimate is limited: The true effect may be substantially different from the estimate of the effect.

Very low certainty: We have very little confidence in the effect estimate: The true effect is likely to be substantially different from the estimate of effect.

^1^There were methodological limitations in randomization and blindness.

^2^The outcomes did not reach the OIS (n = 300) and wide credible intervals suggesting uncertainty in the estimate.

^3^The included studies were performed by the same research group.

## Discussion

This study aimed to assess the effect of O_3_ on color change in tooth bleaching alone and combined with the HP-based bleaching gel, and reduction of tooth sensitivity from the bleaching process in vital teeth. The hypothesis that O_3_ is more effective in the color change in tooth bleaching than HP was rejected, considering that the results between the different methods were statistically similar. It is worth noting that ΔE_Lab_ is an important parameter used to assess the effectiveness of bleaching techniques^39,40^, as values over 1.22 are considered perceptible to the human eye, and color changes over 2.66 are considered acceptable^41,42^. All the studies included in this review reported color change perceptible to the human eye (1.28—1.66)^32^ or acceptable (3.08—6.93)^28–30^ for teeth compared before and after the bleaching therapy. These data are compatible with the studies of laboratory and clinical research^28,33,43–45^. The study by Aykut-Yetkiner and colleagues (2017) presented the lowest ΔE_Lab_ values (1.66 and 1.28), and this is the only study with values classified as perceptible^32^. This result may be related to the older age of patients, which may affect the result of color change in bleaching^40^ compared to the other studies^28–30^.

The bleaching ability has been associated with the oxidative effect of free radicals, released by the breakdown of HP through the formation of hydroxyl and perhydroxyl radicals, superoxide anions, and HP anions, converting the chromophores within hard dental tissues into simpler structures or changing their optical properties. This reflects more light and changes the appearance of the tooth to a lighter shade^3,5,8^. However, a more recent study suggested that HP might whiten normal dentin by oxidizing the benzene ring of aromatic amino acids in dentin phosphoprotein (DPP), which is the main non-collagenous protein located in the organic–inorganic interface and responsible for the fluorescence and color of normal dentin^46^. Moreover, HP can change the translucency property of enamel that became slightly opaquer after bleaching^47^. The O_3_ is an unstable gas that rapidly releases nascent oxygen molecules to form oxygen. Additionally, O_3_ can oxidize the components responsible for tooth discoloration, as chromophore groups may be broken by ozone, forming smaller molecules and resulting in a tooth bleaching effect by one of three mechanisms (bonding mechanism, substitution mechanism, or cleavage mechanism)^28,29^. Both mechanisms seem to have similar bleaching effectiveness, as observed in all studies, because there was no statistical difference between the bleaching techniques and protocols used.

The second hypothesis of the study was rejected. The association of O_3_ with HP does not potentiate the bleaching effect of HP. Although the highest ΔE_Lab_ values were observed in the groups with such association (6.93, 5.85, 5.3), they were not statistically significant in none of the eligible studies. Thus, although O_3_ immediately provides a high amount of OH and O* compounds, such an amount cannot increase the bleaching effect with HP. It is worth noting that the decomposition of HP is slow, so its effectiveness becomes more evident for the in-office technique when at least two clinical sessions are performed^48^. The four eligible studies^28–30,32^ showed that the in-office technique was performed in a single session, showing effective results and clinically perceptible ΔE_Lab_ values. However, the follow-ups were performed in a short time (immediate and 24 and 48 h), which complicates the analysis of the rebound effect^39^ that might show a different response from that obtained in the studies.

Another factor worth mentioning is that three of the eligible studies^28–30^ used HP for 20 min, which is different from the manufacturer's recommendation, and they still obtained acceptable values (3.41, 3.08, 3.15) of color change. Perhaps further studies may be performed to verify whether this reduction in application time might result in bleaching ability similar to the time indicated by the manufacturers, which is usually twice the one used in the eligible studies^12,13^. The reduction of application time would be an important factor that could reduce total chair time and the risk and intensity of tooth sensitivity^12^ because bleaching-induced damage of the dental tissue is cumulative and proportional to the amount of HP that reaches the pulp^10,12,13,49^.

Tooth sensitivity is a major clinical factor that should be considered during and after tooth bleaching, as current studies show that medications used to reduce this painful symptomatology are not effective^3,7,49–51^. The study that used O_3_ before HP showed a perceptible increase in pain sensitivity after bleaching compared with the control group, which leads to the perception that the previous use of O_3_ would both intensify the oxidative power of the gel and increase its diffusion power through the dental tissues, causing pain. Tooth sensitivity is caused by the increase in tooth permeability, changing hydraulic conductance, and dentin intratubular fluid movement, thus providing greater contact between bleaching agents and odontoblastic extensions and pulp tissue, intensifying and providing sensitivity^1–3,6,7,12,13,49^. Two studies described lower sensitivity for the group treated with HP followed by O_3_, while another study described higher sensitivity for the group treated with O_3_ followed by HP. Thus, the order in which the products are applied might be relevant for preventing teeth sensitivity during the bleaching process.

These same studies also show that the use of O_3_ alone does not cause tooth sensitivity as a side effect of whitening and that O_3_ associated and used after HP was effective in preventing such an uncomfortable side effect when using PH in high concentrations^28–30^. This confirms the third and last hypothesis. This factor can be explained by the anti-inflammatory, antioxidant, and analgesic properties of O_3_, which potentially restrict the inflammatory pathways. It has been known that O_3_ is able to neutralize the neurochemical mediators related to pain sensitivity, to inactivate cyclooxygenase by reducing the release of prostaglandins, and to facilitate the metabolization and elimination of inflammatory mediators^1,28–30^.

The side effects resulting from the use of bleaching gels show the need for alternatives that are more biologically compatible with tooth bleaching treatment. Studies reported that the deleterious effects to the dental pulp affected by technique protocol^1,12,13^, gel concentration^49,52^, and secondary components of the bleaching gel formula existent in the commercial product, such as stabilizers, thickeners, dyes, preservatives, and even gel viscosity that reaches the dental pulp might be responsible for affecting the level of diffusion and/or cytotoxicity^8,17^. The manufacturers neither describe nor provide such products.

Our study is not free of limitations, which include some studies performed by the same author, the limited number of RCTs in the literature, the short follow-up period, and the small number of participants per group in the eligible studies. Further studies with a higher number of participants ought to be performed, considering the extensive variability in the ΔE_Lab_ results between the groups (1.28–6.93). Another factor would be the follow-up time, as studies with longer follow-up time would be more interesting, considering there is a difference in the behavior of the values presented in the short and long terms (rebound effect) for the different products in several studies^40,48^. The standardization of time of ozone use is also something to consider because the studies presented different usage periods, ranging from 1^28–30^ to 40^32^ min, without showing differences for the bleaching effect. The last limitation is related to the parameters of color assessment used in the studies because there are current assessment criteria such as WI and ΔE_00_ that are already established in the literature^38^ and considered more perceptive clinically. Such parameters would be ideal to complement the results found in this review, but they could not be calculated because one of the eligible studies did not present isolated L, a, and b values, and they were not even provided by the authors after being contacted via e-mail.

One aspect for consideration in the use of ozone therapy is the need for a financial investment to acquire the ozone generating equipment and the need for caution in handling due to the toxicity of the gas in the respiratory system, which requires technical training before use. However, the equipment would have other clinical uses^18,20–27^ that are not highlighted in this review. The machine allows ozonizing liquids such as water and serum for use in dental procedures, as well as oil^18,19^. During bleaching, although O_3_ did not potentiate the use of HP, it was able to reduce tooth sensitivity to zero, which is one of the greatest challenges and side effects of the technique with HP. Considering such properties and clinical findings for ozone, studies directed to patients presenting clinical conditions considered limiting to conventional tooth bleaching, such as tetracycline staining, tooth sensitivity, and presence of non-carious cervical lesions (NCCL), would be relevant, thus observing their effectiveness and therapeutic clinical response.

### Certainty of evidence and clinical implications

The evidence obtained with this systematic review and meta-analysis was classified as a very low certainty. This result may be explained mainly because of the lack of studies in the literature assessing the use of ozone for bleaching vital teeth. The imprecision found in the pooled estimates reflects the lack of available literature, as the number of participants included in the meta-analysis is one of the factors affecting the confidence interval of the pooled estimates. Moreover, three of the four included studies were published by the same group of researchers (potential risk of publication bias), showing the lack of studies on the topic in other locations in the world. In this context, one way to expand the certainty in estimates regarding the applicability of ozone for vital teeth bleaching is to perform further studies with a higher number of participants by different research groups that comprise different samples.

Other factors that contributed to downgrading the certainty of evidence were methodological limitations and inconsistency among the studies. As in other complementary therapies such as laser therapy, there is still no consensus regarding the optimal protocol for using ozone therapy to bleaching of vital teeth. As a consequence of such a de-standardization, the estimates of the effect of the studies were conflicting. Thus, further studies should establish a protocol of ozone application with strict and adequate methodologies.

Based on the current evidence, the strength of clinical recommendation for the use of ozone therapy for bleaching vital teeth is weak in favor of intervention. This recommendation was based on three main aspects: (1) The low certainty of evidence; (2) The effect estimates of effect found in the meta-analysis were not superior to ozone therapy for any of the outcomes; 3) The cost and investment required for the clinical use of ozone therapy.

Based on limited evidence, the use of O_3_ (alone or associated) was not superior to the conventional use of HP for the bleaching of vital teeth. Moreover, O_3_ cannot intensify the bleaching action of HP, but it showed positive effects for sensitivity.

## Data Availability

The datasets generated during and/or analysed during the current study are available from the corresponding author on reasonable request.
